# Pregnancy Surveillance Methods within Health and Demographic Surveillance Systems

**DOI:** 10.12688/gatesopenres.13332.1

**Published:** 2021-09-13

**Authors:** Christie Kwon, Abu Mohd Naser, Hallie Eilerts, Georges Reniers, Solveig Argeseanu Cunningham

**Affiliations:** 1 Global Health Institute, Emory University, Atlanta, GA, 30322-4201, USA; 2 London School of Hygiene and Tropical Medicine, London, WC1E 7HT, UK

**Keywords:** pregnancy, surveillance, Health and Demographic Surveillance Systems, maternal and child health

## Abstract

**Background:** Pregnancy identification and follow-up surveillance can enhance the reporting of pregnancy outcomes, including stillbirths and perinatal and early postnatal mortality. This paper reviews pregnancy surveillance methods used in Health and Demographic Surveillance Systems (HDSSs) in low- and middle-income countries.

**Methods:** We searched articles containing information about pregnancy identification methods used in HDSSs published between January 2002 and October 2019 using PubMed and Google Scholar. A total of 37 articles were included through literature review and 22 additional articles were identified via manual search of references. We reviewed the gray literature, including websites, online reports, data collection instruments, and HDSS protocols from the Child Health and Mortality Prevention Study (CHAMPS) Network and the International Network for the Demographic Evaluation of Populations and Their Health (INDEPTH). In total, we reviewed information from 52 HDSSs described in 67 sources.

**Results: **Substantial variability exists in pregnancy surveillance approaches across the 52 HDSSs, and surveillance methods are not always clearly documented. 42% of HDSSs applied restrictions based on residency duration to identify who should be included in surveillance. Most commonly, eligible individuals resided in the demographic surveillance area (DSA) for at least three months. 44% of the HDSSs restricted eligibility for pregnancy surveillance based on a woman’s age, with most only monitoring women 15-49 years. 10% had eligibility criteria based on marital status, while 11% explicitly included unmarried women in pregnancy surveillance. 38% allowed proxy respondents to answer questions about a woman’s pregnancy status in her absence. 20% of HDSSs supplemented pregnancy surveillance with investigations by community health workers or key informants and by linking HDSS data with data from antenatal clinics.

**Conclusions:** Methodological guidelines for conducting pregnancy surveillance should be clearly documented and meticulously implemented, as they can have implications for data quality and accurately informing maternal and child health programs.

## Introduction

Reduction in neonatal mortality is a key target of the United Nations’ Sustainable Development Goals ^1^. While the global under-five mortality rate has declined by almost two thirds over the past 30 years ^2^, neonatal mortality has declined more slowly. Efforts to accelerate the pace of reduction are hindered by a lack of accurate and timely data on child deaths in the regions where they are most prevalent. Such information is crucial to guiding resource allocation and evaluating the effectiveness of interventions ^3, 4^. In countries with high infant mortality in South Asia and Sub-Saharan Africa, estimates of neonatal mortality based on health care utilization data or clinic reports exclude women and children who do not have access, do not seek care, or die at home ^5^. The United Nations Inter-agency Group for Child Mortality Estimation (UN IGME) stillbirth and child mortality estimates are derived from vital registration systems, censuses, and various demographic health survey data ^6^. Poor data quality in many developing nations makes accurate estimation more challenging ^7^. Furthermore, the use of models to correct such estimates is complicated by lingering questions regarding their applicability to African settings ^8^.

Pregnancy surveillance is in theory an important tool in the identification of stillbirths and neonatal deaths and could circumvent some of the methodological flaws of retrospective reports in cross-sectional surveys ^3^. The term pregnancy detection commonly refers to activities that only identify pregnancy, without the follow-up tracking of pregnancies through to their end. Pregnancy surveillance entails activities to identify pregnancies and monitor their outcomes. The latter is often integrated into health and demographic surveillance systems (HDSSs), which collect population-based data about pregnancies and maternal and child health ^3^. HDSSs conduct continuous registration of demographic and vital events in a geographically defined surveillance area (DSA) in low- and middle-income countries ^9^. HDSS fieldworkers conduct regular visits to collect demographic and health data from all households under surveillance. Fieldworkers generally collect pregnancy data for all eligible women as part of these regular visits.

The goal of this report is to describe the methods used to conduct population-based pregnancy surveillance low- and middle-income countries. We investigate variation in pregnancy surveillance methods across HDSSs and identify the components that may be linked with data completeness and quality in capturing stillbirths and neonatal deaths. This approach expands the limited literature analyzing pregnancy surveillance methodologies ^3, 10^ and draws on protocols and information from within and outside of the published literature.

## Methods

We reviewed published literature, HDSS field manuals and data collection instruments for methods used in pregnancy identification and follow-up ( Table 1). Articles published between 2002 and 2019 in English were searched in PubMed and Google Scholar using the search terms: pregnan*, identif*, discover*, population, surveillance, observation, registration, detection, demograph*, maternal, surveys and questionnaires, INDEPTH Network, epidemiology, DHS, DSS, and HDSS.

**Table 1.** Search terms used for identification of papers pertaining to pregnancy surveillance.

Search terms in PubMed	Initial results	Considered for review *	Included in review **
(identification or identify or discover or discovery) and ("Population Surveillance"[MeSH] or surveillance[tw]) AND ("Pregnancy"[MeSH] or pregnant or pregnancy) And ("Surveys and Questionnaires"[MeSH] or questionnaire[tw] or survey[tw])	682	15	6
(HDSS profile) and ("Population Surveillance"[MeSH] or surveillance[tw]) AND ("Pregnancy"[MeSH] or pregnant or pregnancy) And ("Surveys and Questionnaires"[MeSH] or questionnaire[tw] or survey[tw])	3	3	0
HDSS Profile	34	26	24
“INDEPTH Network”	595	24	2
(("Population Surveillance/methods"[MAJR]) AND "Pregnancy"[MeSH Terms]) AND epidemiology and INDEPTH	6	5	0
(demographic surveillance sites) AND (pregnancy OR maternal)	894	20	0
(("Pregnancy"[MeSH]) AND "Population Surveillance"[MeSH]) AND "Surveys and Questionnaires"[MeSH] AND "Demography"[MeSH]	1866	5	0
**Search terms in Google Scholar**			
pregnancy AND "INDEPTH Network" AND HDSS	659	44	5
DHS and (DSS or HDSS) and pregnancy	127	5	0
**Total articles**	4866	**147**	**37**

* Based on review of titles and abstracts

** Based on main text review and removal of duplicates

Abbreviations: HDSS = health and demographic surveillance systems; [MeSH] = Medical Subject Headings is the NLM controlled vocabulary thesaurus used for indexing articles for PubMed; [tw] = Text Words includes all words and numbers in the title, abstract, other abstract, MeSH terms, MeSH Subheadings, Publication Types, Substance Names, Personal Name as Subject, Corporate Author, Secondary Source, Comment/Correction Notes, and Other Terms when searching in PubMed

**Figure 1.**
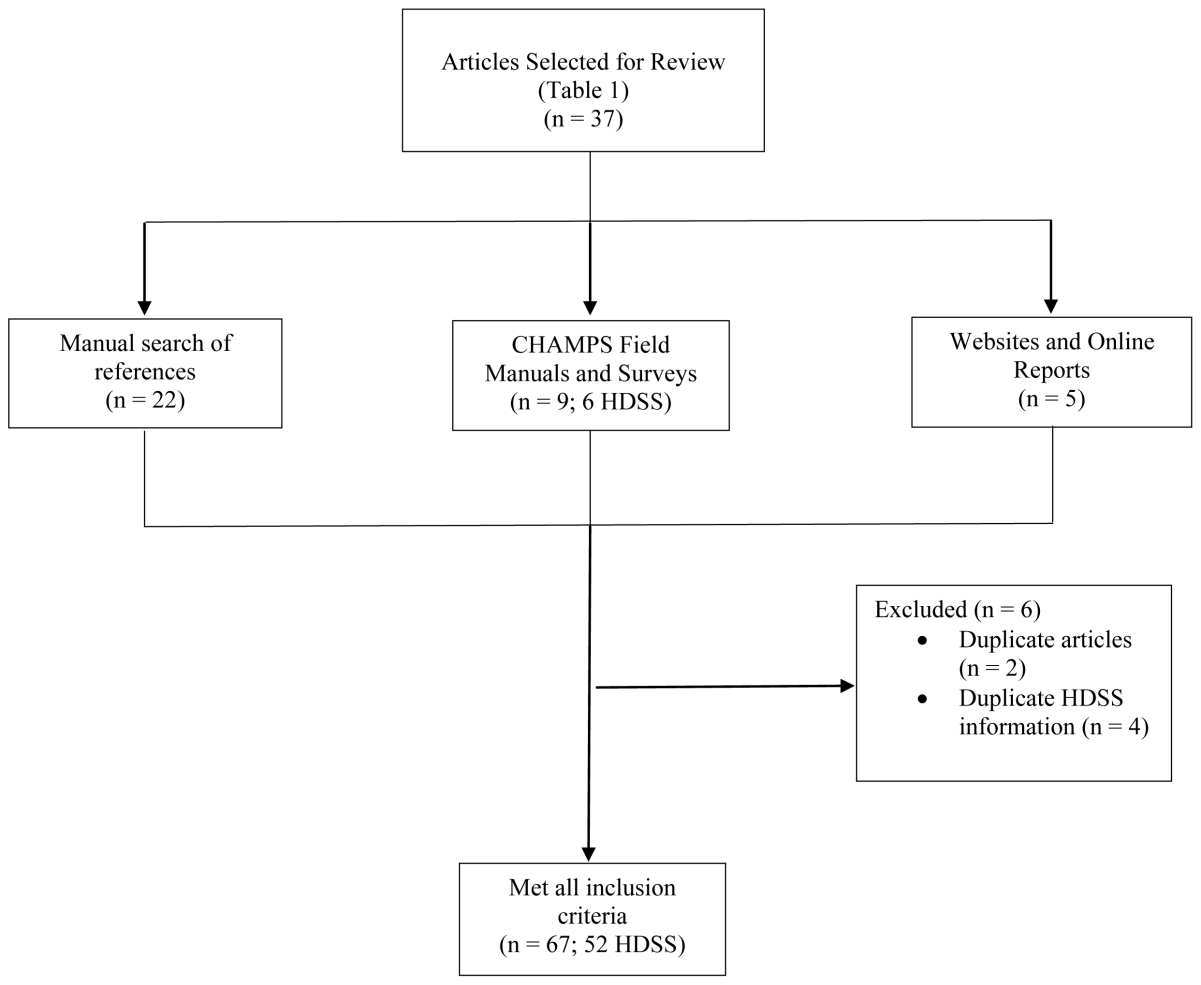
Literature review flowchart.

Following a review of titles and abstracts, we reviewed the main text of 147 articles mentioning pregnancy-related data collection in HDSSs. In total, 37 articles reporting any information about data collection methods or criteria for pregnancy surveillance were retained. An additional 22 articles were located through a manual search of the references of included studies. We also reviewed the websites of two networks: the Child Health and Mortality Prevention Study (CHAMPS) Network is a network of HDSSs in six countries that are focused on identifying the main causes of under-5 mortality (Cunningham, 2019); the International Network for the Demographic Evaluation of Populations and their Health (INDEPTH) is a network of 49 independent HDSSs and 7 associate HDSSs in Africa, Asia, and Oceania ^11^, for online data reports, data collection instruments and field protocols of HDSSs within CHAMPS and INDEPTH. Collectively, we gathered information from 67 sources ( Figure 1).

The review included 52 HDSSs located in 20 countries, which represent the majority of operating sites. For each, we reviewed the criteria used to define women who are eligible for HDSS pregnancy surveillance, requirements for respondents from whom pregnancy information can be collected, visit frequencies for collecting pregnancy information, questions asked for pregnancy identification, supplemental efforts for enhancing pregnancy surveillance beyond regular HDSS household visits, and rules regarding characteristics of interviewers administering pregnancy-related questionnaires.

## Results

### Eligibility criteria of women to be included in pregnancy surveillance

Each HDSS sets criteria for who is considered part of the population under surveillance. The eligible population is determined by residency within the geographically defined area or membership in a household under surveillance. Residency length is the number of months a woman has lived continuously within the DSA. Of the 52 HDSSs, 24 (46%) reported required residency lengths between three and six months for a woman to be considered part of the population under surveillance; more than half of sites did not report a residency eligibility threshold ( Table 2).

**Table 2.** Information about pregnancy surveillance methodology from 52 health and demographic surveillance systems (HDSSs).

HDSS	Eligibility criteria			Use of proxy respondents					Data collection frequency (months)	Supplemental surveillance activities ^b^
	*Residence* * requirement* * (months)*	*Marital* * status ^a^*	*Age * *range*	*Head of * *household*	*Spouse*	*Adult* *member*	*Adult * *women/ * *mothers*	*Any * *household * *member*		
**Asia**										
Baliakandi, Bangladesh ^9^	4	Y	< 50	N	N	N	N	N	2	
Chakaria, Bangladesh ^30^	6	Y	15–49						3	
Matlab, Bangladesh ^13^		Y	15–49	N	N	N	Y	N	2	
Ballabgarh, India ^3^									1	
Birbhum, India ^31^	6	N							0.5	
Muzaffarpur-TMRC, India ^22^	6								2	Y ^f^
Vadu, India ^3, 29^			d	N	N	Y	N	N	6	Y
DodaLab (Hanoi), Vietnam ^36^									3	
FilaBavi, Vietnam ^36^									3	
South East Asia Community Observatory (SEACO) ^37^	3									
**Africa**										
Dande, Angola ^38^	3 ^c^								12	
Kaya, Burkina Faso ^3^				N	N	N	N	Y	6	
Nanoro, Burkina Faso ^3, 39^	3								4	
Nouna, Burkina Faso ^40^									4	
Ouagadougou, Burkina Faso ^3, 17^	6	N	15–49	N	N	Y	N	N		
Taabo , Coˆte d’Ivoire ^3, 26^	4		12–55	N	N	Y	N	N	4	
Butajira, Ethiopia ^3^				Y	Y	Y	N	N	3	
Dabat, Ethiopia ^3^				N	N	N	Y	N	6	Y
Gilgel Gibe, Ethiopia ^3^				Y	Y	Y	N	N	6	
Kersa/Harar, Ethiopia ^9, 23^	6	Y	14–49	Y	N	Y	Y	N	6	
Kilte Awulaelo, Ethiopia ^13^			15–49						6	
Farfenni, Gambia ^3, 11, 41^									3	
Kiang West, Gambia ^33^									3	
Dodowa, Ghana ^3, 14^									6	
Kintampo, Ghana ^3^				Y	Y	Y	Y	Y	12	Y
Navrongo, Ghana ^3^									4	
Bandim, Guinea-Bissau ^13, 25^			13–49	Y	Y	Y	Y	Y	1 - urban; 6 - rural	Y
Kilifi, Kenya ^3, 20^	3 ^c^	N	13–55						4	
Kisumu {KEMRI/CDC), Kenya ^28^	4		<15, 15-55 ^d^	Y	Y	Y	Y	Y	4	Y ^f^
Kombewa, Kenya ^3, 42^									6	Y
Manyatta/Siaya-Karemo, Kenya ^9^	4		<15, 15-55 ^d^	Y	Y	Y	Y	Y	6	
Mbita, Kenya ^3, 43^									3	
Nairobi, Kenya ^3^				Y	N	Y	N	N	4	
Rusinga, Kenya ^44^									4	
Siaya, Kenya ^3^									4	
Karonga, Malawi ^3, 45^									12	Y ^e^
Bamako, Mali ^9^	6 ^c^	N	14–49	N	N	N	N	N	6	Y
Manhica, Mozambique ^9, 46^	3 ^c^		12–49						6	Y ^f^
Nahuche, Nigeria ^12^	3		15–49						6	
Bandafassi, Senegal ^19^			13–55						12	
Mlomp, Senegal ^18^			13–55						12	
Niakhar, Senegal ^47^	6								4	
Africa Centre, South Africa ^3^				Y	N	N	N	N	4	
Agincourt, South Africa ^3, 21^	6	N	12–49	Y	Y	Y	N	N	12	
Dikgale, South Africa ^27^	6									
Soweto, South Africa ^9^	4		15–49						6	
Dar Es Salaam, Tanzania ^16^	3		15–49						6	
Ifakara, Tanzania ^3, 48^	4								4	
Magu, Tanzania ^15^	3 ^c^	N	15–49							
Rufiji, Tanzania ^3, 24^	4	Y	13–49						4	
Iganga Mayuge, Uganda ^13^			15–49	Y	Y	Y	Y	N	6	Y ^e^
Kyamulimbwa, Uganda ^3^				Y	N	N	Y	N	12	

Note: This table presents a review of sites from the articles identified as part of this review. Data are based on the most current published information. Superscripts in column 1 indicate the sources of information for each row. Symbols indicate the following: (Y) required or permitted; (N) not required or not permitted.

^a^Includes married or unmarried women in pregnancy surveillance specifically mentioned

bIncludes data collection supplemented by community key informants, scouts, village healthworkers, clinic data

^c^Eligibility based on planned months to reside rather than actual residence period

^d^Child-bearing age, including <15 years of age

^e^Paid $1-3

^f^Aligned to health center or antenatal clinic data

HDSS protocols also define who is eligible for pregnancy surveillance. Surveillance is typically conducted for those considered “at risk” of becoming pregnant as defined by age and marital status. In total, 44% of the HDSS used age cutoffs for pregnancy surveillance: nine asked pregnancy-related questions only of women ages 15–49 years ^3, 9, 12– 17^; three of women ages 13–55 years ^3, 18– 20^; two each of women ages 12–49 years ^3, 9, 21, 22^, 14–49 years ^9, 23^, and 13–49 years ^3, 13, 24, 25^; and one of women ages 12–55 years ^3, 26^. Four HDSS had more ambiguous age cutoffs, with one including women under age 50 years ^9^, and three sites including all women of ‘child-bearing age’ without further specification ^3, 27– 29^. A total of 21% of HDSSs considered marital status as a criterion for inclusion in the pregnancy surveillance, of which five (45%) included only married women in pregnancy surveillance ^3, 9, 13, 23, 24, 30^, and the other six sites (55%) explicitly mentioned inclusion of both unmarried and married women ^3, 9, 15, 17, 20, 21, 31^.

### Pregnancy surveillance key indicators

Across HDSS, a variety of pregnancy identification and pregnancy history questions are asked ( Table 3). Three of the six CHAMPS sites directly ask about current pregnancy; the other three ask indirectly through questions about the date of her last menstrual period or by asking if a woman has ever been pregnant, currently or in the past. The most common indicators used across HDSSs are: (1) whether the woman is currently pregnant; (2) number of months pregnant; (3) expected delivery date based on last menstrual date; (4) whether the pregnancy was confirmed by ultrasound or pregnancy test; (5) whether the woman had antenatal care during this pregnancy; and (6) information about previous pregnancies, including pregnancy loss, termination, stillbirths and live births.

**Table 3.** Overview of typical pregnancy surveillance data elements collected.

Pregnancy detection	
*Current pregnancy*	• Is <Name>/are you pregnant (now)? ¥
*Expected delivery date*	• What is the expected date of delivery? ¥ Ѫ • When was your (her) last menstrual period (date)? § Ѱ • What is the estimated date of conception? Θ • Approximate number of months pregnant? Ѫ
*Pregnancy confirmation*	• Was the pregnancy confirmed by any of the following? Ѱ *Pregnant belly visually apparent;* *Pregnant woman has felt fetal movement;* *Positive pregnancy test at home;* *Physical exam;* *Auscultation of fetal heart tones;* *Ultrasound;* *Positive pregnancy test at a facility;*
*Follow-up*	• From today's date, what is the current status? Ѫ *Still pregnant;* *Already delivered;* *Had an abortion;* *Had a stillbirth*
**Pregnancy history**	
*Past pregnancies*	• Have you/Has the woman ever been pregnant (including the current)? Ѱ • How many times have you/she been pregnant, including livebirth, stillbirth and miscarriages/ abortions? Ѱ • Have you given birth in the past 4 weeks? § • How many alive children do you have? §
*Past births*	• While pregnant have you/has the woman ever given birth? Ѱ • How many live births have you had in your life? Ѫ • I would like to ask if you gave birth to any children in the last 5 years. How many? Ѯ • Are you less than 6 months postpartum or fully breastfeeding and free from menstrual bleeding since you had your child? §
*Past losses*	• How many times have you had pregnancies that resulted in a miscarriage/unexpected abortion (abortion=less than 20 weeks of gestation)? Ѫ • Number of miscarriages or stillbirths (including the current), if any. Ѫ • Have you had miscarriage or abortion the past 7 days? § • Have you ever given birth to a boy or girl who was born alive but later died before age 5 years? Ѯ
*Other*	• Have you been using a reliable contraceptive (pills, injectable, IUCD and Norplant) method consistently and correctly? § • How many pregnant women slept in this household yesterday? Ѫ

¥ Siaya-Karemo and Manyatta HDSSs, Kenya

§ Kersa and Harar HDSSs, Ethiopia

Ѫ Manhiça HDSS, Mozambique

Ѱ Baliakandi HDSS, Bangladesh

Ѯ Soweto and Thembelihle HDSSs, South Africa

Θ INDEPTH Network Sample Pregnancy Registration Form ( http://www.indepth-network.org/Resource%20Kit/INDEPTH%20DSS%20Resource%20Kit/LinkedDocuments/Sample%20DSS%20Pregnancy%20Registration%20Form.pdf)

### Proxy respondents

Of the 52 HDSSs, 38% specified who could report on a woman’s pregnancy status ( Table 2). Two only allowed pregnancy-related questions to be asked of a woman herself ^9^, while 18 HDSSs allowed proxy respondents: 5 allowed any household member ^3, 9, 13, 25, 28^, non-specific for age but often preferring to ask those >15 years of age; 13 allowed proxy reports from any adult household member over 18 years of age ^3, 9, 13, 17, 21, 23, 25, 26, 28, 29^; 8 from husbands ^3, 9, 13, 21, 25, 26, 28^; 12 from the household head ^3, 9, 13, 21, 23, 25, 28^; and 9 from any other adult women or mothers ^3, 9, 13, 23, 25, 28^. 

### HDSS data collection intervals and supplementary surveillance methods

Across HDSSs, the median interval between household visits to collect information, including on pregnancies, is four months. Most HDSS conduct visits between three and six months, but some had visits as frequently as every two weeks or as infrequently as once every 12 months ( Table 2). The frequency of visits has varied over time in many sites due to changes in funding and research goals.

In total, 20% of the HDSSs reported supplemental pregnancy surveillance efforts, in addition to standard demographic surveillance. A common approach is to have trained community health workers (CHWs) conducting additional home visits to record new pregnancies and pregnancy outcomes between regular interview rounds ^32^. CHWs are used at sites in Karonga, Malawi ^3^; Dabat, Ethiopia; Bandim, Guinea-Bissau; and Kintampo, Ghana ^32^. At some sites, HDSS data are linked with electronic medical records and automatically updated when women in the HDSS visit an antenatal care clinic ^33^. Other HDSSs maintain continuous community reporting for demographic events between regular DSS rounds ^28^. In such systems, trained community interviewers update record births, deaths, and pregnancies as they occur in the community, using mobile electronic devices between HDSS rounds. This method limits the number of events that are not reported or reported with delays ^10, 28^. At some sites, community informants or HDSS supervisors visit antenatal clinics and community health centers daily or weekly to gather information about pregnancies and deliveries of HDSS residents ^34^.

### Interviewer characteristics

Depending on the HDSS, routine surveillance visits are conducted by teams of field workers and supervisors, or trained, local residents. Interviewers are required to have completed a certain level of education, such as high school education in Dabat, Ethiopia; secondary education in Gilgel Gibe, Ethiopia; and O level certificate of education in Nairobi, Kenya ^3, 35^. In general, community scouts and informants must be >15 years of age. HDSSs in Dabat, Ethiopia and Matlab, Bangladesh require that community informants be married ^35^; Matlab HDSS additionally stipulates that interviewers must be female. For the HDSS in Nahuche, Nigeria, male fieldworkers are not allowed to interview females ^12^. DodaLab also employs primarily female field surveyors to conduct all HDSS surveillance, including for questions related to pregnancy surveillance ^36^.

## Discussion

HDSSs provide reference data for health and demographic estimates in countries without civil registration and vital statistics systems; they complement periodic cross-sectional censuses and surveys. Pregnancy surveillance is an important component of health and demographic surveillance activities. The prospective follow-up of pregnancies can produce better estimates of stillbirths and early childhood mortality. We document that the pregnancy surveillance methods used in HDSS are heterogeneous. Methods differ in the eligibility criteria for women to be included in the pregnancy surveillance, the frequency of visit intervals, the use of proxy respondents, and the characteristics required of interviewers. The methods employed can affect the quality and completeness of information on pregnancies and their outcomes. Surveillance methods are not always described in detail, making comparability even more difficult.

HDSS residency requirements determine which women are part of the population under surveillance and are particularly important in areas where migration occurs frequently ^32^. The definition of “residents” can affect the size of the population under surveillance, as well as the capturing of demographic events. Populations located in urban areas that experience high mobility can be more difficult to track through temporary migration patterns. In some communities, it is common for women to temporarily relocate to their parents’ home for the months before and after the birth of a child ^49, 50^. A recent in-migrant may not be immediately recorded as a resident in the HDSS, in which case her pregnancy would not be recorded in that visit. If she is still present in the household in the next round of household visits and meets the residency criterion, she will be recorded and so will her child; however, if the child did not survive to the next HDSS round, the child may not be listed onto the household roster and will thus not be counted. A solution for HDSS with high rates of migration has been to create a temporary residency status, which allows women to be included in surveillance even if it is unclear whether they will become permanent residents.

Many HDSS have restrictions on the marital status and ages of women for whom pregnancy information is collected. These are generally useful, but that can sometimes exclude some women who have a chance to be pregnant from being recorded. It is inappropriate in some communities to ask unmarried women about pregnancy status, and unmarried women are less likely to report their pregnancies overall ^13, 51^. Also women who are very young or at older ages may not disclose pregnancies ^52^. This is due to the sensitive nature of pregnancy information. Unmarried women may be more vulnerable to stigma or not ready to disclose an out-of-wedlock pregnancy ^53^. There may be shame around having an unplanned pregnancy; for women who are at risk of pregnancy loss due to their age, they may want to avoid the shame that can accompany pregnancy loss, or being suspected of having terminated the pregnancy ^53^. Unmarried women are likely to be young and at higher risk of adverse pregnancy outcomes ^54^. Never-married women under age 25 account for 3.5–60% of births in sub-Saharan Africa ^55^. Thus, pregnancies and especially pregnancies with high risk of adverse outcomes can be missed. 

Pregnancy surveillance inclusion criteria may need to be designed with the higher risk of negative birth outcomes for older, younger, and unmarried women in mind. Broader inclusion for pregnancy surveillance may improve the tracking and detection of possible adverse birth outcomes for women who might not be counted otherwise. An important consideration for electronic data collection is the preprogrammed rules that set data validation cutoffs in the system based on age or marital status. Overly restrictive rules may impede data collection. For example, if the data collection device does not allow interviewers to record pregnancies for women under the age of 15, or pregnancies to unmarried women, such information will not be registered even if offered. Allowing interviewers to record out of range values makes it possible to record such information. 

If the respondent is not home when an interviewer visits, many HDSSs allow for information to be collected from another person in the household, to reduce the need for re-visits. However, especially for sensitive information related to pregnancy, proxy responses may not be accurate. Use of proxy respondents is an important contributor to the undercounting of pregnancies, especially in the first two trimesters when women tend not to discuss a pregnancy with others ^10^. Pregnancy, stillbirth and early neonatal death are often hidden because they increase women’s vulnerability to social and physical harm through gossip, blame, acts of violence, or beliefs about sorcery and spiritual possession ^52^. Therefore, proxy respondents may not be aware of another individual’s pregnancy or feel comfortable sharing such information, leading to underreporting. Even though it is more time-consuming, it is best to only ask women themselves about their own pregnancies. If women are absent at the time of visit, interviewers should return at least twice to speak with her directly before using proxy respondents ^32^. Additionally, many HDSS are now using telephone and text to reach respondents, and these modes may also be appropriate for contacting women about pregnancies. 

Because pregnancies only last up to nine months, infrequent data collection increases the risk of missing pregnancies altogether ^15, 38^. The more frequent the HDSS visits, the more likely that pregnancies will be recorded. When pregnancies are missed, the outcomes of pregnancies are often also not recorded. This is especially true for pregnancies that ended in stillbirth or neonatal death. Data collection less than 5 months apart are recommended ^32^. Additionally, more frequent visits can build community engagement and rapport when inquiring about sensitive subjects such as pregnancy. The CHAMPS network recommends visiting each catchment area every few months to improve tracking of both pregnancy and migration-- the latter of which serves to improve tracking of temporarily absent women ^9^.

Supplemental pregnancy surveillance activities can provide useful avenues for improving pregnancy detection. Linking health facility data to HDSS increases data when pregnancies are missed by the regular data collection and they can be recorded retrospectively after birth from hospital or health center data ^35^, though only for women who access healthcare. Additionally, networks of informants can supplement HDSS capacity by providing alerts about vital events as they occur, including pregnancy. 

Interviewers need to be well trained to collect information in sensitive situations. In some settings, interviewers from outside the community may be better positioned to collect sensitive information. For information regarding pregnancies, female interviewers are highly recommended. Women are more likely to disclose pregnancies to other women ^36, 56^. One study found that interviewers who were female, younger, and conducted more interviews elicited more responses on a survey about social networks, on average ^56^. Abortion and stillbirth are also less likely to be reported to interviewers who are men ^10^. While the impact of interviewer gender on other components of HDSS data collection is inconclusive, the use of female interviewers is preferred for collecting pregnancy data because female respondents disclose more sexual behavior related information to same-gender interviewers ^57, 58^. Cultural norms in Nahuche demand the use of predominantly female interviewers and have resulted in higher quality data and lower refusal rates ^59^.

The pregnancy surveillance methods in HDSS are diverse and often lack detailed documentation. There are also questions surrounding the quality of HDSS estimates of neonatal mortality, which are subject to huge variability and are on average lower than corresponding estimates from demographic health surveys ^7^. Unreliable data on the vital events of newborns has been described as one of the most challenging issues facing HDSS ^60^. Children who survive are likely to be recorded by the HDSS when they are observed in subsequent interview rounds. However, those that die are more easily missed, especially in the absence of a pregnancy notification. Pregnancy surveillance facilitates the follow-up of adverse pregnancy outcomes and early mortality. The inclusion criteria for pregnancy surveillance, respondent and interviewer characteristics, and the frequency of household visits should be considered further for their potential impact on the identification of pregnancies and their follow-up. Improvements to HDSS pregnancy surveillance protocols and completeness have the potential to address well-documented issues of downward bias in early mortality estimates. The standardization of pregnancy reporting protocols and exhaustive capture of pregnancy status information should thus be among the highest priorities for HDSSs.

## Data availability

All data underlying the results are available as part of the article and no additional source data are required.
