# Supplementary material for: Pregnancy Surveillance Methods within Health and Demographic Surveillance Systems
Source: Gates Open Res. 2021 Sep 13;5:144. [Version 1] doi: 10.12688/gatesopenres.13332.1 (PMC8960731; doi:10.12688/gatesopenres.13332.1)
Supplement: Peer review history

# Peer review: Reviewer response for version 1

**DOI:** 10.21956/gatesopenres.14575.r31701

**Published:** 2022-03-28

**Authors:** Sangappa M. Dhaded

**Affiliations:** 1 Women's and Children's Health Research Unit, JN Medical College, KLE Academy of Higher Education and Research Belagavi, Belagavi, Karnataka, India

**Introduction:**

 Pregnancy surveillance systems are an important aspect of public health. The manuscript addresses a pregnancy surveillance system which is necessary to know pregnancy outcome and plan intervention. It also points to lacunae in current methods used for pregnancy surveillance, indicating the need for methods to obtain reliable data.

**Methods:**

 Reviewed literature published over the last 17 years and other sources like field manuals, data collecting instruments. Focuses on parameters related to pregnancy surveillance systems like women eligibility, specification for respondent, visit frequency to collect data and questions to identify pregnancy.

**Results:**

 Presentation of results is appropriate. It includes various aspects of pregnancy surveillance systems as per reviewed literature. Variables are eligibility criteria for women, indicators of pregnancy, proxy respondent, data collection interval and characteristics of interviewer. 

**Discussion:**

 It is based on the results of literature search, and highlights the importance of HDSS as a complimentary to census and surveys. The heterogenous nature of pregnancy surveillance systems is highlighted in relation to women eligibility, visit frequency, proxy respondent and interviewer characteristics. 

 It also states about incomplete description of surveillance methods.

**Conclusion:**

 Appropriate as it highlights the diverse nature and lack of detailed documentation in surveillance systems in addition to data quality issues. Thus emphasizing the need for standardization of pregnancy outcome reporting systems/protocols.

Is the work clearly and accurately presented and does it cite the current literature?

Yes

If applicable, is the statistical analysis and its interpretation appropriate?

Not applicable

Are all the source data underlying the results available to ensure full reproducibility?

Yes

Is the study design appropriate and is the work technically sound?

Yes

Are the conclusions drawn adequately supported by the results?

Yes

Are sufficient details of methods and analysis provided to allow replication by others?

Yes

Reviewer Expertise:

I am a neonatologist by training, working in medical school as a teacher and a consultant neonatologist in in a affiliated hospital. I have also experience in implementation of research projects to improve maternal and newborn health for the last 17 years.

I confirm that I have read this submission and believe that I have an appropriate level of expertise to confirm that it is of an acceptable scientific standard.

# Peer review: Reviewer response for version 1

**DOI:** 10.21956/gatesopenres.14575.r31700

**Published:** 2022-03-07

**Authors:** Claudia Hanson

**Affiliations:** 1 Department of Public Health Sciences, Karolinska Institutet, Solna, Sweden

This paper reviews pregnancy surveillance methods used in Health and Demographic Surveillance Systems (HDSSs) in low- and middle-income countries. The research included papers published in PubMed complemented by a Google Scholar search. A total of 37 articles were included through literature review and 22 additional articles were identified via manual search of references. The different ways how pregnancies are assessed were presented tabular. This is a well written paper, providing an important overview. The methods are well explained. My only comment is that I would recommend adding on how the outcomes of pregnancy are attained – thus abortion, miscarriage, stillbirth, livebirth. The discussion is appropriate.

Is the work clearly and accurately presented and does it cite the current literature?

Yes

If applicable, is the statistical analysis and its interpretation appropriate?

Not applicable

Are all the source data underlying the results available to ensure full reproducibility?

No source data required

Is the study design appropriate and is the work technically sound?

Yes

Are the conclusions drawn adequately supported by the results?

Yes

Are sufficient details of methods and analysis provided to allow replication by others?

Yes

Reviewer Expertise:

NA

I confirm that I have read this submission and believe that I have an appropriate level of expertise to confirm that it is of an acceptable scientific standard.
